# Kalirin, a Key Player in Synapse Formation, Is Implicated in Human Diseases

**DOI:** 10.1155/2012/728161

**Published:** 2012-04-03

**Authors:** Prashant Mandela, Xin-Ming Ma

**Affiliations:** Department of Neuroscience, University of Connecticut Health Center, Farmington, CT 06030, USA

## Abstract

Synapse formation is considered to be crucial for learning and memory. Understanding the underlying molecular mechanisms of synapse formation is a key to understanding learning and memory. Kalirin-7, a major isoform of Kalirin in adult rodent brain, is an essential component of mature excitatory synapses. Kalirin-7 interacts with multiple PDZ-domain-containing proteins including PSD95, spinophilin, and GluR1 through its PDZ-binding motif. In cultured hippocampal/cortical neurons, overexpression of Kalirin-7 increases spine density and spine size whereas reduction of endogenous Kalirin-7 expression decreases synapse number, and spine density. In Kalirin-7 knockout mice, spine length, synapse number, and postsynaptic density (PSD) size are decreased in hippocampal CA1 pyramidal neurons; these morphological alterations are accompanied by a deficiency in long-term potentiation (LTP) and a decreased spontaneous excitatory postsynaptic current (sEPSC) frequency. Human Kalirin-7, also known as Duo or Huntingtin-associated protein-interacting protein (HAPIP), is equivalent to rat Kalirin-7. Recent studies show that Kalirin is relevant to many human diseases such as Huntington's Disease, Alzheimer's Disease, ischemic stroke, schizophrenia, depression, and cocaine addiction. This paper summarizes our recent understanding of Kalirin function.

## 1. Kalirin Is a Rho Guanine Nucleotide Exchange Factor (GEF)

Kalirin was discovered 15 years ago as a novel protein that interacts with the cytosolic carboxyl-terminal of peptidylglycine *α*-amidating monooxygenase (PAM), an integral membrane peptide processing enzyme [1]. We have made significant progress in understanding the functions of Kalirin; like the many other Rho-GEFs encoded in mammalian genomes, Kalirin promotes the exchange of GDP for GTP and thus stimulates the activity of specific Rho GTPases [2, 3]. Rho GTPases that regulate multiple cellular processes play a key role in transducing signals from extracellular stimuli to the intracellular pathways that play a pivotal role in the formation of dendritic spines and synaptic development [4–6].

## 2. Multiple Kalirin Isoforms

**Figure 1:**
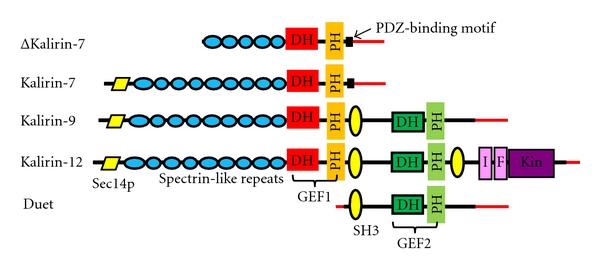
Major Kalirin isoforms. Alternative splicing generates different isoforms of Kalirin. DH: Dbl homology; PH: pleckstrin homology; GEF1: guanine nucleotide exchange factor 1; SH3: Src homology domain; GEF2: guanine nucleotide exchange factor 2; I: immunoglobulin-like; F: fibronectin III-like; Kin: kinase domain; red lines, unique 5′- and 3′-untranslated regions.

**Figure 2:**
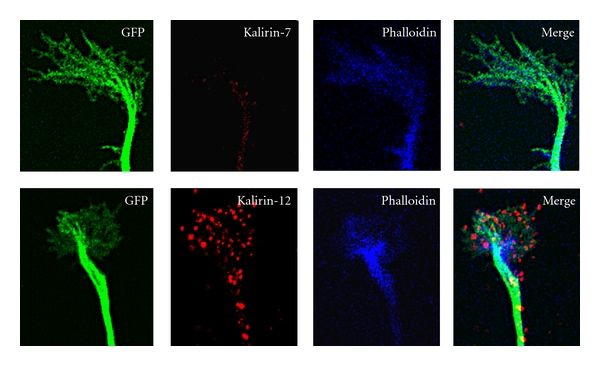
Kalirin-12, not Kalirin-7, is localized to the growth cone of hippocampal neurons. Primary cultures of hippocampal neurons prepared at embryonic day 20 (E20) were transfected with a vector encoding GFP on the day of culture preparation as described [19]. On day 3 after transfection, cultures were fixed for staining of filamentous actin with Alexa Fluor 633 Phalloidin (Life Technologies) and endogenous Kalirin-7 or Kalirin-12 with isoform-specific rabbit antibodies. Rabbit antibodies were visualized using Cy3 donkey anti-rabbit IgG (The Jackson Laboratory). Images were collected with a Zeiss confocal microscope LSM510.

The mouse Kalirin gene (*Kalrn*) consists of 65 exons spanning >650 kb of the genome; the presence of multiple promoters and transcriptional start sites enables the production of multiple functional isoforms of Kalirin [7–9]. Each Kalirin isoform is composed of a unique collection of domains (Figure 1). Major Kalirin isoforms including Kalirin-7, -9, and -12 are generated through the use of alternative 3′ exons [8]. The major isoforms share some common features including nine spectrin-like repeats, the GEF1 domain and the Sec14p domain. Sec14p domains facilitate lipid interactions and cellular localization. The nine spectrin-like repeat regions that follow the Sec14p domain have been shown to interact with many proteins including disrupted in schizophrenia 1 (DISC1) [10], peptidylglycine *α*-amidating monooxygenase (PAM) [1], inducible nitric oxide synthase (iNOS) [11], Huntingtin-associated protein 1 (HAP1) [12], and Arf6 (ADP-ribosylation factor 6) [13]. Kalirin-12 is the longest isoform and contains additional domains that include GEF2, an immunoglobulin-like (Ig) domain, a fibronectin III (FnIII) domain, and a serine/threonine protein kinase domain that is followed by a short, unique carboxyl-terminus [7, 14]. Kalirin-12 is found in the growth cones of immature neurons (Figure 2) and dendritic spines of mature cultured hippocampal neurons, suggesting a role for Kalirin-12 in axon outgrowth and synaptic plasticity. Interaction of the Ig-FnIII region unique to Kalirin-12 with the GTPase domain of dynamin may facilitate the coordination of endocytic trafficking and changes in the actin cytoskeleton [15]. Endogenous Kalirin-9 is localized to neurites and growth cones, and expression of exogenous Kalirin-9 induces longer neurites and altered neuronal morphology in cultured cortical neurons [16]. The functions of Kalirin-9 and Kalirin-12 in neurons remain to be elucidated. The ΔKalirin-7 (also referred as Kalirin-5) isoform is generated using a different promoter, and translation initiation begins at the start of spectrin-like repeat 5, producing an isoform with only 5 spectrin-like repeats. Overexpression of ΔKalirin-7 results in an increase in spine size, but not spine density, in cultured cortical neurons [17]. Duet, an isoform of Kalirin that begins just before the second GEF domain and continues through the unique C-terminal of Kalirin-12, uses a third promoter [7]. Duet was discovered as a protein homologous to the catalytic domain of death associated protein kinase and is localized to actin-associated cytoskeletal elements, suggesting the involvement of Duet in cytoskeleton-dependent functions [18].

## 3. Tissue Expression

Kalirin expression is detectable in a wide array of adult tissues including neurons, endocrine cells, liver, muscle, and heart [2, 20, 21]. In addition, developmentally regulated, tissue specific-Kalirin isoform expression is evident. Kalirin-9 and -12 are highly expressed in neuronal tissue during embryonic development, while in the adult brain expression of each is drastically decreased; a concomitant increase in Kalirin-7 expression occurs [8, 20, 22]. Kalirin-7 expression is largely limited to neurons of the central nervous system; its levels are extremely low at birth (postnatal days 1–7) and begin to increase markedly at postnatal day 14, which coincides with the onset of maximum synaptogenesis [22, 23].

## 4. Kalirin-7 Is the Major Kalirin Isoform in Adult Brain

Kalirin-7 is the most abundant Kalirin isoform in the adult rodent brain and is exclusively localized to the postsynaptic side of excitatory synapses [19, 22, 39, 40]. Kalirin-7 is a multifaceted molecule containing domains that interact with a wide array of molecular machinery. The Sec14p domain located at the N-terminus interacts with phosphatidylinositol-3-phosphate and plays a key role in Kalirin-7-mediated spine morphogenesis (Ma et al., unpublished). The C-terminus of Kalirin-7 contains a unique PDZ-binding motif through which Kalirin-7 interacts with PDZ-domain-containing proteins including PSD-95, AF-6, and spinophilin [39]. Binding of the NMDA receptor subunit NR2B to the PH domain of Kalirin-7 is important for normal synaptic plasticity [41]. The spectrin-like domains of Kalirin-7 through which it interacts with DISC1, iNOS, PAM, HAP1, and Arf6 play a key role in Kalirin-7-induced synapse formation (Ma et al., unpublished). The nucleotide sequences of human Kalirin-7 (Duo) and rat Kalirin-7 are 91% identical, and their amino acid sequences are 98% identical; human Kalirin-7 contains a 27-nucleotide insert not found in rat Kalirin-7, located at the end of the region encoding the seventh spectrin repeat [8, 12].

## 5. Kalirin-7 Contains Multiple Phosphorylation Sites

Phosphorylation of Kalirin-7 has recently been shown to be a pivotal mechanism mediating Kalirin-7-induced spine formation and synaptic plasticity [42–44]. Purified PKA, PKC, CaMKII, Cdk5, and Fyn each phosphorylate purified Kalirin-7 [45]. Kalirin-7 is extensively phosphorylated *in vivo*. The phosphorylation sites identified *in vitro* using purified CaMKII, PKA, PKC, or CKII, identified only 5 of the 22 sites that undergo phosphorylation in cells or tissue. These findings emphasize a critical role for additional protein kinases and the importance of cellular localization in the phosphorylation of Kalirin-7 [45]. Densely distributed phosphorylation sites have been identified in the spectrin-like repeat region, many lying on the fourth and fifth repeats. Their phosphorylation state could influence on a wide array of protein-protein interactions that involve this region. Since the first four spectrin-like repeats are absent in ΔKalirin-7 (Figure 1), the phosphorylation sites in the missing region could play a key role in the differences in spine size and density associated with expression of full length Kalirin-7 versus ΔKalirin-7. Overexpression of exogenous Kalirin-7 results in an increase in both spine density and spine size while overexpression of ΔKalirin-7 only increases spine size without altering spine density [17]. Although phosphorylation sites that regulate GEF activity as assessed in intact cells have been identified, a relationship between phosphorylation state and GEF activity has not yet been demonstrated using purified proteins.

## 6. Kalirin-7 Plays a Key Role in Spine/Synapse Formation *In Vitro*

Kalirin-7 expression is limited to neurons in the CNS [22, 30, 46]. Immunostaining in cultured hippocampal and cortical neurons demonstrates that Kalirin-7 clusters are apposed to glutamate-transporter-1-(Vglut1-) positive clusters, a marker for excitatory presynaptic terminals [19, 47, 48]. When cultured neurons are fixed with cold methanol, it can be seen that the Kalirin-7 positive clusters consistently overlap clusters positive for PSD95, NMDA receptor subunits NR1 and NR2B, or AMPA receptor subunits GluR1 and GluR2; overlap is apparent in both dendritic spines and in the dendritic shaft [19, 40]. In contrast, Kalirin-7 clusters neither align with GAD65-positive clusters, a marker for inhibitory presynaptic terminals, nor with GABA_A_ receptor positive clusters, a marker for inhibitory postsynaptic endings [19]. These findings lead to the conclusion that Kalirin-7 is localized almost entirely to postsynaptic excitatory terminals.

**Figure 3:**
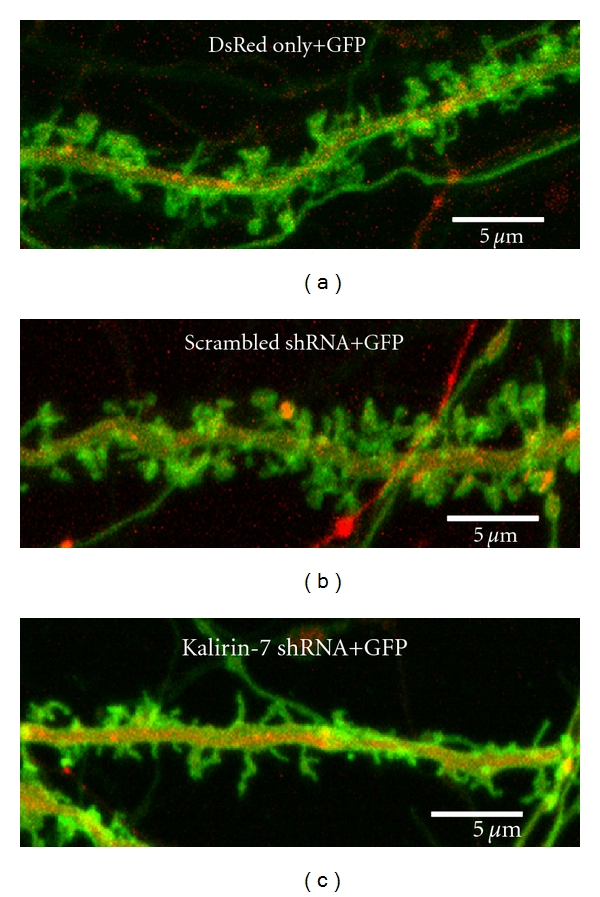
Expression of Kalirin-7 shRNA causes a reduction in spine density and spine size in cultured hippocampal neurons. Cultured hippocampal neurons prepared at E20 were transfected with vector encoding a membrane-tethered version of GFP (pmGFP) alone [(a), spine density 8.9 ± 0.8/10 *μ*m], pmGFP plus a scrambled shRNA [(b), spine density 9.2 ± 0.9/10 *μ*m], or pmGFP plus pSIREN-Kalirin-7 shRNA [(c), spine density 5.2 ± 0.6/10 *μ*m] on the day of culture preparation. The specificity of pSIREN-Kalirin-7 shRNA was determined previously [19]. DsRed marks transfected neurons expressing the shRNA. Images were collected on day 20 with an LSM510 confocal microscope. Expression of Kalirin-7 shRNA, not scrambled shRNA, caused a 80% decrease in Kalirin-7 staining (not shown). Spine density was determined using Metamorph (Molecular Devices, Downington, PA) as described [54]. Scale bar = 5 *μ*m.

Regulation of Kalirin-7 expression by synaptic activity in hippocampal neurons suggests that Kalirin-7 plays a pivotal role in the regulation of excitatory synapse formation and signaling [40, 42]. Overexpression of Kalirin-7 causes an increase in dendritic spine density, spine size, and synapse number, while reduction of endogenous Kalirin-7 levels leads to a reduction in spine density, spine size, and synapse number in cultured hippocampal and cortical neurons [19, 22, 39] (Figure 3). Interestingly, overexpression of Kalirin-7 induces spine formation in spine-free hippocampal interneurons [19] and a recent study reports an important role of Kalirin-7 in regulating dendrite growth in cortical interneurons [44]. A delicate balance between synaptic excitation and inhibition is critical for maintaining normal circuits in the CNS, and interneurons play an essential role in regulating local circuit excitability [49]. Understanding Kalirin-7 function in interneurons may increase our understanding of neurological diseases such as epilepsy, bipolar disorder, schizophrenia, autism, and Alzheimer's Disease, which are related to disruption of GABAergic interneuron development [50–53].

## 7. Kalirin-7 Plays a Key Role in Spine/Synapse Formation *In Vivo*

Two *Kalrn* knockout mouse models have been developed: the Kalirin-7 knockout mouse (Kalirin-7^KO^) [54] and the GEF1 Kalirin knockout mouse (Kalirin^GEF1-KO^) [55]. Kalirin-7^KO^ mice, in which the terminal exon unique to Kalirin-7 was deleted, grow and reproduce normally. Hippocampal CA1 pyramidal neurons of Kalirin-7^KO^ mice show a 15% decrease in spine density and deficits in long-term potentiation (LTP). Morphological alterations in Kalirin-7^KO^ mice are accompanied by behavioral alterations including decreased anxiety-like behavior in the elevated zero maze and impaired acquisition of a passive avoidance task. Kalirin-7^KO^ mice exhibit normal behavior in the open field, object recognition, and radial arm maze tasks [54]. PSDs purified from the cortices of Kalirin-7^KO^ mice show a deficit in Cdk5, a kinase known to phosphorylate Kalirin-7 and play an essential role in Kalirin-7-mediated spine formation and synaptic function [43, 54, 56]. Furthermore, NR2B levels are modestly decreased in PSD preparations from the cortices of Kalirin-7^KO^ animals [54]. This decrease is accompanied by decreased levels of NR2B-dependent NMDA receptor currents in cortical pyramidal neurons [41]. NR2B plays a critical role in LTP induction, dendritic spine formation, different forms of synaptic plasticity, learning, and memory [57–60]. Decreased NR2B levels may partially contribute to decreased spine density and the deficit in LTP induction observed in Kalirin-7^KO^ mice. Importantly, expression of exogenous Kalirin-7 in cultured Kalirin-7^KO^ neurons rescues decreased spine density. These findings show that Kalirin-7 plays an essential role in synaptic structure and function.

The Kalirin^GEF1-KO^ mice were generated by replacing exons 27-28 in the first GEF domain by a neomycin resistance cassette, thus eliminating production of the major Kalirin isoforms; in addition to Kalirin-7, these mice are unable to produce Kalirin-9 and Kalirin-12 [55]. Kalirin^GEF1-KO^ mice show significant morphological deficits including reduced size of both cortex and decreased spine density in pyramidal neurons of the cortex [55]. Interestingly, in Kalirin^GEF1-KO^ mice the hippocampus is reduced in size but spine density in hippocampal neurons is normal. This reduction in hippocampal size in Kalirin^GEF1-KO^ mice results from neuronal loss since Kalirin is exclusively expressed in pyramidal neurons, granule cells of the dentate gyrus, and interneurons scattered throughout the hippocampus [19, 22, 46, 54]. Both Kalirin-7^KO^ and Kalirin^GEF1-KO^ mice show impaired hippocampal LTP induction, impaired contextual fear conditioning, and reduced spine density in cultured cortical neurons and normal long-term memory [54, 61]. Kalirin^GEF1-KO^ mice show deficits in working memory and an intact reference memory. However, Kalirin-7^KO^ and Kalirin^GEF1-KO^ mice exhibit distinct differences in their behavioral phenotype. Kalirin^GEF1-KO^ mice show very high locomotor activity in the open field and a deficit in spatial memory, which are not affected in Kalirin-7^KO^ mice [54, 55]. Further comparisons of the phenotypes of the Kalirin^GEF1-KO^ and Kalirin-7^KO^ mice are needed, along with the generation of additional *Kalrn* knockout mouse models generated using different knockout strategies.

## 8. Kalirin-7 Is Implicated in Cocaine Addiction

Cocaine addiction is a chronic relapsing neurological disorder associated with severe medical and psychosocial complications [62–66]. The long-lasting nature of cocaine addiction leads to relapse and makes it especially difficult to treat [21]. Repeated cocaine treatments increase dendritic spine density/spine head size and neurite complexity in the brain's reward circuitry such as the medium spiny neurons (MSNs) of the nucleus accumbens (NAc). Our study shows that Kalirin-7 is an essential determinant of dendritic spine formation following cocaine treatment [38]. Kalirin-7 is expressed in the MSNs of the NAc, a key area in the brain involved in drug addiction and reward pathways [37]. Chronic cocaine treatment of wild-type mice results in an increase in Kalirin-7 expression in the NAc which is accompanied by an increase in spine density in the MSNs of NAc core [37, 67]. This cocaine-induced increase in dendritic spine density in the NAc MSNs in wild-type mice is abolished in Kalirin-7^KO^ mice. Both wild-type and Kalirin-7^KO^ mice have identical spine densities in the MSNs of the NAc prior to cocaine treatment. These morphological changes could underlie the behavioral variations seen in these mice following cocaine treatment. Chronic cocaine treatment leads to increased locomotor sensitization in Kalirin-7^KO^ mice compared to wild-type controls [38]. These data suggest Kalirin-7 plays an important role in the mechanism of cocaine addiction, which needs to be addressed in the future.

## 9. Kalirin Is Implicated in Human Diseases

Altered Kalirin expression has been reported in several neuropsychiatric, neurological and cardiovascular diseases as well as animal models of depression, epilepsy and cocaine addiction (Table 1). Genetic analyses have identified *KALRN* as a major risk factor in stroke and early onset of coronary artery disease [34–36]. Similarly in schizophrenia, decreased dendritic spine density in the prefrontal cortex is reported to correlate with decreased Kalirin mRNA levels [24]. A rare missense mutation in the *KALRN* gene has been shown to be a genetic risk factor for schizophrenia [26]. The spectrin-like repeat region of Kalirin has also been shown to interact with DISC1, a genetic risk factor for schizophrenia which plays an important role in activity-dependent spine elongation by promoting Kalirin-7/Rac-1 interactions [10, 24, 68, 69]. Attention-deficit/hyperactivity disorder (ADHD) is the most common neurobehavioral disorder and the underlying molecular mechanisms of ADHD are largely unknown. Animal models of ADHD are associated with spine loss in striatal MSNs [70] and functional impairments in glutamatergic synaptic transmission in the hippocampus [71]. A genomewide association study of ADHD patients has also implicated alterations in Kalirin expression in ADHD [33]. Dendritic pathology and decreased dendritic spine density are prominent phenomena in early cases of Alzheimer's Disease, which correlate significantly with the progressive decline of mental faculties [72–75]. Alzheimer's Disease patients also show a significant decrease in Kalirin mRNA and protein expression in the hippocampus without significant changes in other brain regions [28]. This decrease in Kalirin expression has been associated with increased iNOS activity both in hippocampus from Alzheimer's patients and in cultured neuroblastoma cells [27]. These data lead to the hypothesis that lack of Kalirin is associated with the dendritic alterations and substantial decrease in spine density observed in Alzheimer's Disease.

The cause underlying major depression and the neurobiological basis of antidepressant therapy are not clear. Altered synaptic plasticity may play a key role in the pathogenesis and treatment of depression [76]. Major depression is associated with spine reductions in the hippocampus [77]. Kalirin expression and spine density in the hippocampus are decreased in the animal models of depression [29, 31, 78]. Kalirin levels and spine density in the hippocampal CA1 pyramidal neurons increase after repeated electroconvulsive treatment (ECT) [30, 79], one of the most effective therapies for depression [80, 81]. These observations suggest a role for Kalirin in the development of depression. In epileptic patients and in experimental models of epilepsy, there is a marked spine loss, abnormal spine shape, and alteration in dendritic morphology in hippocampal CA1 pyramidal neurons [82, 83]. Animal models of epilepsy show a marked increase in Kalirin expression in the hippocampus [32], suggesting a role for Kalirin in the neuropathology of human epilepsy. Kalirin interacts with Huntingtin-associated protein 1 (HAP1) [12] and HAP1 dysfunction could contribute to the selective neuropathology in Huntingtin's disease [84]. Huntingtin's disease is characterized by loss of medium-sized spiny neurons in the striatum [85], alterations in spine density, and abnormalities in the size and shape of dendritic spines in striatal MSNs [86–88]. Overexpression of Kalirin-7 caused an increase in spine density while reduced expression of Kalirin-7 resulted in loss of dendritic spines and a decrease in dendritic complexity in the MSNs of striatal slice cultures which mimic *in vivo* conditions (Ma et al., unpublished). These data raise the possibility that Kalirin-7 may play a role in the neuropathology of Huntington's disease [87]. Finally, Kalirn-7 plays an important role in estrogen-mediated spine/synapse formation in the hippocampus [31, 40]. Regulation of Kalirin-7 by estrogen suggests a role for Kalirin in ovarian hormone-associated cognitive function and menopause-associated disorders [89–91]. Taken together, altered dendritic spine morphology and spine density remain the hallmarks of many human neurological and psychiatric disorders. There is a correlation between the levels of Kalirin expression and the pathology of dendritic spines in some psychiatric and neurological disorders. It is important to understand whether Kalirin is a trigger or one of many factors mediating the dendritic spine pathology of these diseases, which may be rescued by altering Kalirin expression/function or targeting downstream signals of Kalirin.

## 10. Conclusion/Future Studies

Kalirin-7, the major isoform of Kalirin in the adult brain, plays a critical role in spine formation/synaptic plasticity. Estrogen-mediated spine formation in hippocampal neurons and cocaine-induced increases in spine density in striatal MSNs require Kalirin-7. Kalirin has also been implicated in many neuropsychiatric and neurological diseases. Future studies will focus on investigating the underlying molecular mechanisms of Kalirin-7-mediated spine formation/synaptic plasticity and its role in neuropsychiatric and neurological diseases.

## Figures and Tables

**Table 1:** Kalirin and human diseases.

Kalirin isoform	Disease	Physiological relevance	References
Kalirin-7 (Duo)	Schizophrenia	Decreased spine density, decreased Kalirin-7 mRNA levels in the prefrontal cortex	[24]
Kalirin (unknown isoforms)	Schizophrenia	Kalirin mRNA increases 2-fold in the prefrontal cortex	[25]
Kalirin-7	Schizophrenia	DISC1 regulates spine formation via Kal7-Rac1	[10]
Kalirin	Schizophrenia	Kalirin is a risk factor for schizophrenia	[26]
Kalirin-7	Alzheimer's Disease	Decreased levels of Kalirin-7 mRNA and protein in hippocampus	[27, 28]
Kalirin (unknown isoforms)	Animal model of depression	Decreased Kalirin expression in the prefrontal cortex and hippocampus. ECT increases Kalirin expression in hippocampus	[29, 30]
Kalirin-7	Animal model of depression	Decreased Kalirin-7 expression in hippocampus	[31]
Kalirin (unknown isoforms)	Animal model of epilepsy	Decreased Kalirin expression in hippocampus	[32]
Kalirin (unknown isoforms)	ADHD	Unknown	[33]
Kalirin (unknown isoforms)	Stroke	Unknown	[34, 35]
Kalirin (unknown isoforms)	Coronary Heart disease	Unknown	[36]
Kalirin (unknown isoforms)	Huntington's disease	Spectrin-like domains of Kalirin interact with HAP1	[12]
Kalirin-7	Animal model of cocaine addiction	An essential determinant of dendritic spine formation following cocaine treatment	[37, 38]
